# Inhibition of Src kinase activity attenuates amyloid associated microgliosis in a murine model of Alzheimer’s disease

**DOI:** 10.1186/1742-2094-9-117

**Published:** 2012-07-02

**Authors:** Gunjan Dhawan, Colin K Combs

**Affiliations:** 1 Department of Pharmacology, Physiology and Therapeutics, University of North Dakota, Grand Forks, ND, 58203, USA; 2 School of Medicine and Health Sciences, 504 Hamline St., Room 118, Grand Forks, ND, 58203, USA

## Abstract

**Background:**

Microglial activation is an important histologic characteristic of the pathology of Alzheimer’s disease (AD). One hypothesis is that amyloid beta (Aβ) peptide serves as a specific stimulus for tyrosine kinase-based microglial activation leading to pro-inflammatory changes that contribute to disease. Therefore, inhibiting Aβ stimulation of microglia may prove to be an important therapeutic strategy for AD.

**Methods:**

Primary murine microglia cultures and the murine microglia cell line, BV2, were used for stimulation with fibrillar Aβ1-42. The non-receptor tyrosine kinase inhibitor, dasatinib, was used to treat the cells to determine whether Src family kinase activity was required for the Aβ stimulated signaling response and subsequent increase in TNFα secretion using Western blot analysis and enzyme-linked immunosorbent assay (ELISA), respectively. A histologic longitudinal analysis was performed using an AD transgenic mouse model, APP/PS1, to determine an age at which microglial protein tyrosine kinase levels increased in order to administer dasatinib via mini osmotic pump diffusion. Effects of dasatinib administration on microglial and astroglial activation, protein phosphotyrosine levels, active Src kinase levels, Aβ plaque deposition, and spatial working memory were assessed via immunohistochemistry, Western blot, and T maze analysis.

**Results:**

Aβ fibrils stimulated primary murine microglia via a tyrosine kinase pathway involving Src kinase that was attenuated by dasatinib. Dasatinib administration to APP/PS1 mice decreased protein phosphotyrosine, active Src, reactive microglia, and TNFα levels in the hippocampus and temporal cortex. The drug had no effect on GFAP levels, Aβ plaque load, or the related tyrosine kinase, Lyn. These anti-inflammatory changes correlated with improved performance on the T maze test in dasatinib infused animals compared to control animals.

**Conclusions:**

These data suggest that amyloid dependent microgliosis may be Src kinase dependent *in vitro* and *in vivo.* This study defines a role for Src kinase in the microgliosis characteristic of diseased brains and suggests that particular tyrosine kinase inhibition may be a valid anti-inflammatory approach to disease. Dasatinib is an FDA-approved drug for treating chronic myeloid leukemia cancer with a reported ability to cross the blood-brain barrier. Therefore, this suggests a novel use for this drug as well as similar acting molecules.

## Introduction

Amyloid beta deposition and microglial activation are two major pathophysiologic hallmarks of the progression of Alzheimer’s disease, often suggested to be associated with each other [1]‐[9]. Microglia are the resident phagocytes of the central nervous system. In the AD brain, microglia are found in a highly activated state often in association with senile plaques [10]‐[13]. Activated microglia present a different phenotype as compared to resting microglia, and are responsible for, in particular, pro-inflammatory cytokine secretions [10]‐[18]. Aβ accumulation in AD has been associated with these increases in pro-inflammatory markers [19]‐[25]. Aβ peptide has the ability to self-aggregate to form oligomers and fibrils. Both forms have been reported to have neurotoxic and gliotic actions *in vitro* as well as *in vivo*[24]‐[36]. Because of the close proximity of reactive microglia to deposited fibrils of Aβ, it is hypothesized that Aβ acts as a stimulus for microglial activation during disease to initiate or propagate the inflammatory changes observed. Therefore, there is a need to identify a therapeutically approachable microglial target for reducing inflammation in the brains of AD patients. Based upon the fact that recent data demonstrate that prolonged non-steroidal anti-inflammatory drug use decreases incidence of AD when taken by asymptomatic individuals, an anti-inflammatory approach to disease may be an effective prevention strategy [37]. It is imperative to understand the mechanism(s) by which microglia become reactive to better design anti-inflammatory drug strategies.

Although data suggest a causative role of Aβ deposition for microgliosis, the underlying mechanisms involved are not fully resolved [10,18,28,38]‐[41]. It has been demonstrated through a variety of studies that Aβ is capable of stimulating microglia *in vitro* and *in vivo* to increase protein phosphotyrosine levels. This correlates well with the reported increase in microglial phospho-tyrosine immunoreactivity in AD brains [42]. These data have supported a hypothesis that the increase in phosphotyrosine immunoreactivity is due to either increased tyrosine kinase activity or decreased tyrosine phosphatase activity. It appears that both scenarios may be true. Microglia can use a multi-receptor complex for interacting with Aβ fibrils on the plasma membrane [43]. Upon ligand binding, a specific signaling pathway is activated involving propagation and downstream increased activation of numerous non-receptor tyrosine kinases, including Src, Lyn, FAK and PYK2 [24,28,39,41,44]‐[47]. Based upon inhibition studies, the increased tyrosine kinase enzyme activities upon Aβ binding are absolutely critical for microgliosis to occur [24,28,39,41,44]‐[47]. In fact, it appears that not only increased tyrosine kinase activity is required for Aβ stimulation but also decreased tyrosine phosphatase activity [48,49]. Our prior work demonstrated that both oligomeric and fibrillar forms of Aβ stimulate increased protein phosphotyrosine levels *in vitro* and *in vivo* that correlated with activation of non-receptor tyrosine kinases [38,50]. Irrespective of the form of Aβ involved, one common mechanism of action appears to be involvement of tyrosine kinases leading to increased secretion of proinflammatory cytokines. This study tests whether inhibition of the Aβ fibril-stimulated signaling response, more precisely non-receptor tyrosine kinase activity, can attenuate microgliosis both *in vitro* and *in vivo*. The Src-Abl inhibitor, dasatinib, was used to treat primary murine microglia cultures *in vitro*. In order to quantify effects of dasatinib in a more physiologically relevant form of disease, the drug was also administered to a transgenic mouse model of AD. This APP/PS1 mouse line expresses a Swedish mutation in APP and a deltaE9 mutation of presenilin 1 (PS1). The mice over-express human Aβ with a correlating high Aβ plaque immunoreactivity and microgliosis [51,52].

In this work, we demonstrated using primary murine microglia cultures that dasatinib was able to attenuate the Aβ-dependent increase in overall protein phospho-tyrosine levels and active levels of Src and Lyn non-receptor tyrosine kinases which correlated with decreased TNFα secretion. In addition to the in vitro analyses, dasatinib was able to reduce active Src but not Lyn levels as well as TNFα and microgliosis in the APP/PS1 mice following 28 days of subcutaneous infusion. Our study indicates that attenuation of specific non-receptor tyrosine kinase activities, in our case using an FDA approved cancer drug, dasatinib, may be therapeutically useful as a novel anti-inflammatory approach to AD.

## Methods

### Materials

Anti-Aβ, clones 6E10 and 4G8 were from Covance (Emeryville, CA, USA). The anti-Lyn antibody, anti-Src, anti-α-tubulin antibodies, FITC and Texas Red conjugated secondary antibodies, and horseradish peroxidase conjugated secondary antibodies were purchased from Santa Cruz Biotechnology (Santa Cruz, CA, USA). Mouse TNF-α Elisa kit was obtained from R&D Systems (Minneapolis, MN, USA). Anti-phosphotyrosine (4G10) antibody was from EMD Millipore (Billerica, MA, USA) and anti-pLyn (Tyr 396) antibody was purchased from Abcam (Cambridge, MA, USA). Anti-Iba1 antibody was from Wako Chemicals USA, Inc (Richmond, VA, USA). Elite Vectastain ABC avidin and biotin and alkaline phosphatase kits, biotinylated anti-rabbit, anti-mouse, and anti-rat antibodies and the Vector VIP and Vector Blue chromogen kits were from Vector Laboratories Inc. (Burlingame, CA, USA). Anti-CD68 was obtained from Serotec (Raleigh, NC, USA). Anti-PSD95 and anti-pSrc (Tyr416) antibodies were purchased from Cell Signaling Technology (Danvers, MA, USA). Anti-APP antibody was purchased from Invitrogen (Camarillo, CA, USA). Synaptophysin and βIII tubulin antibodies were purchased from Chemicon International, Inc (Temecula, CA, USA). The non-receptor tyrosine kinase inhibitor, dasatinib, was obtained from LC Laboratories (Woburn, MA, USA). The transgenic mouse line, strain 005864 B6.Cg-Tg(APPswe,PSEN1dE9)85Dbo/J and wild type mouse line, C57BL/6 were obtained from the Jackson Laboratory (Bar Habor, ME, USA).

### Animal use

All animal use was approved by the University of North Dakota Institutional Animal Care and Use Committee (UND IACUC). Mice were provided food and water *ad libitum* and housed in a 12 h light:dark cycle. The investigation conforms to the National Research Council of the National Academies Guide for the Care and Use of Laboratory Animals (8^th^ edition).

### BV2 cell line

Immortalized murine microglial BV2 cells were obtained from Dr. Gary E. Landreth, Cleveland, OH, USA. The cells were maintained at 3 × 10^6^ cells/dish in 100-mm dishes in DMEM/F12 (Gibco RBL, Rockville, MD, USA) supplemented with 10% heat-inactivated fetal bovine serum (FBS) (U.S. Biotechnologies Inc., Parkerford, PA, USA), 5% horse serum (Equitech-Bio, Inc., Kerrville, TX, USA), penicillin G (100 units/ml), streptomycin (100 mg/ml), and L-glutamine (2 mM) and incubated at 37°C in a humidified atmosphere containing 5% CO_2_ and 95% air.

### Murine microglia culture

Microglia were derived, as described previously [53], from the brains of postnatal day 1 to 3 C57BL/6 J mice. Briefly, meninges-free cortices were removed, trypsinized and triturated in microglia media (DMEM/F12 media containing L-Glutamine (Invitrogen, Carlsbad, CA, USA) and 20% heat inactivated FBS) and placed in T-75 flasks. The media in the flasks was replaced completely after 24 h and partially after 7 days with fresh media. The cells were ready to harvest and count after 14 days.

### Cell stimulation

Aβ 1–42 fibrils (American Peptide, Sunnyvale, CA, USA) were prepared according to an established protocol [54]. Microglia were stimulated by removing them from growth media into serum-free DMEM/F12 media containing Aβ fibrils. To assess dasatinib effects, BV2 or microglia were pretreated with the drug for 30 minutes before Aβ stimulations. For Western blot analyses, five- minute Aβ stimulations were performed and cells were lysed using radioimmunoprecipitation assay buffer (RIPA) (20 mM Tris, pH 7.4, 150 mM NaCl, 1 mM Na3VO4 10 mM NaF, 1 mM EDTA, 1 mM EGTA, 0.2 mM phenylmethylsulfonyl fluoride, 1% Triton X-100, 0.1% SDS, and 0.5% deoxycholate) with protease inhibitors (AEBSF 104 mM, Aprotinin 0.08 mM, Leupeptin 2.1 mM, Bestatin 3.6 mM, Pepstatin A 1.5 mM, E-64 mM). Protein concentrations were determined using the Bradford method [55]. For ELISA and toxicity analyses, cells were stimulated with Aβ for 24 hours. To assess the level of proinflammatory cytokine, TNF-α, secreted after 24 h stimulation of microglia with Aβ fibril, the media from the cells was collected and analyzed using a commercially available ELISA kits (R & D Systems) according to the manufacturer’s protocol. Cell viability was assessed by the MTT reduction assay. After 24-hour stimulation of microglia, media was removed for ELISA and the cells were incubated with 3[4,5-dimethylthiazol-2-y1]-2,5-diphenyltetrazolium bromide (MTT, 100 μg/mL) for 4 hours. The media was aspirated and the reduced formazan precipitate was dissolved in isopropanol. Absorbance values were read at 560/650 nm via plate reader and averaged +/− SEM.

### Western blot analysis of microglia cultures

Lysates from cell stimulation experiments were diluted into sample buffer and separated via 10% SDS-PAGE, transferred to polyvinylidene difluoride membrane and Western blotted using anti-pSrc (Tyr416), anti-pLyn (Tyr396), Src (loading control) and Lyn (loading control) and enhanced chemiluminescence for detection (GE Healthcare, Piscataway, NJ, USA). pSrc/pLyn optical densities (O.D.) from visualized Western blots were normalized to their respective loading controls (Src/Lyn) and averaged from five independent experiments.

### Collection of brains from different age APP/PS1 mice

Brains from different aged APP/PS1 mice were collected for longitudinal analyses. 2-, 4-, 6- and 12-month old transgenic mice (n = 5 to 6) along with their age-matched C57BL/six wild type controls were euthanized and perfused with PBS-CaCl_2_. Brains were rapidly dissected and divided into left and right hemispheres, with right hemispheres fixed in 4% paraformaldehyde for sectioning. The left hemispheres were further dissected into different brain regions to obtain hippocampus, temporal cortex, frontal cortex and cerebellum, and flash frozen using liquid N_2_. The frozen tissue was lysed using RIPA with protease inhibitors and used for Western blot analysis.

### Subcutaneous infusions of dasatinib into APP/PS1 mice

Dasatinib was infused subcutaneously into female APP/PS1 mouse at 13 months of age. Dasatinib was delivered via mini-osmotic pumps (model 1004, 0.25 μL/hour delivery rate, Alzet, Cupertino, CA, USA). Pumps delivered either vehicle (DMSO/Hepes) (n = 6) or dasatinib (500 ng/kg/day) (n = 7) for 28 days. At the end of the infusion period, mice were euthanized, brains perfused with PBS-CaCl_2_ and rapidly collected. Control untreated APP/PS1 animals were collected at a comparable age of completion, 14 months. The right hemispheres were collected for fixing in 4% paraformaldehyde and the left hemispheres were flash frozen in liquid nitrogen for biochemical analysis.

### Immunostaining mouse brains

The paraformaldehyde fixed right hemispheres for different age mice or from dasatinib treated and control mice were cut using a freezing microtome. Briefly, paraformaldehyde fixed tissue was embedded in a 15% gelatin (in 0.1 M phosphate buffer) matrix and immersed in a 4% paraformaldehyde solution for two days to harden the gelatin matrix. The blocks were then cryoprotected through three cycles of 30% sucrose for three to four days each. The blocks were then flash frozen using dry ice/isomethylpentane, and serial 40 μm sections were cut using a freezing microtome. Serial sections were used for immunostaining with anti- pTyr (4G10) antibody at a dilution of 1:1,000, anti-Aβ (4G8) and anti-CD68 at a dilution of 1:500 to 1:1,000, anti-pSrc as 1:250, anti-Iba1 at 1:1,000, and anti-GFAP antibody at a dilution of 1:1,000, followed by incubation with biotinylated secondary antibodies (1:2,000 dilution) (Vector Laboratories Inc.) and avidin/biotin solution (Vector ABC kit). The immunoreactivity was observed using Vector VIP as chromogen. For pSrc/CD68 double staining, FITC and Texas Red conjugated secondary antibodies were used. For phosphotyrosine/CD68 or Iba1 double-labeling, the sections were first immunostained with anti-phosphotyrosine antibody 4G10 using Vector VIP as the chromogen. The tissue was then incubated in 0.2 N HCl to strip off antibodies then the tissue was double-labeled with either CD68 or Iba1 using Vector Blue as the chromogen. The slides were dehydrated and cover slipped using VectaMount (Vector Laboratories, Inc.) following a standard dehydrating procedure through a series of ethanol solutions and Histo-Clear (National Diagnostics, Atlanta, GA, USA). Images were taken using an upright Leica DM1000 microscope and Leica DF320 digital camera system (Leica Microsystems Inc., Buffalo Grove, IL, USA). Figures were made using Adobe Photoshop 7.0 software (Adobe Systems, San Jose, CA). For quantitation purposes, 1.25X images were taken from three consecutive serial sections, (960 μm apart) throughout the hippocampal region. Optical densities from the temporal cortex or CA1 regions from the same serial sections were measured using Adobe Photoshop software. All sections were immunostained simultaneously to minimize variability and background values in an unstained area of tissue for each section were set to zero using the curve tool before quantifying optical density values. The optical density of the entire temporal cortex region/CA1 region from a representative section was selected via marquee and the same size marquee was applied to all sections per condition to allow comparison of optical densities (O.D.) independent of area changes. The values for each section were averaged (three sections/brain, five to seven brains per condition) and plotted for Aβ, immunoreactivity for dasatinib infusion animals, pSrc immunoreactivities of dastinib treated mice, and Aβ and CD68 immunoreactivities for longitudinal study animals. For quantitating phospho-tyrosine immunostaining from different aged APP/PS1 and wild type mice, the serial sections were viewed under a microscope and the number of 4G10 positive plaques were viewed from the entire CA1 and temporal cortex regions for all the animals in each condition. The numbers of plaques were averaged (three sections/brain, five to seven brains per condition) and plotted. To insure reliability in comparison, only immunostains that were processed together were quantitatively compared to minimize any variability in staining processing from day-to-day. This allows an accurate assessment of relative comparisons within parallel processed conditions and samples although not necessarily a reflection of absolute values.

### Western blot analyses of mouse brains

Hippocampus and temporal cortex were removed from flash frozen brains of treated mice, lysed, sonicated in RIPA buffer and quantitated using the Bradford method [55]. The lysates were resolved using a custom-built 28-well comb and 10% SDS-PAGE and transferred to polyvinylidene difluoride membranes for Western blotting using anti-pTyr (4G10), anti-APP, anti-Aβ (6E10), anti-GFAP, anti-TNF-α and anti-CD68 antibodies with α-tubulin as their loading control, anti-pSrc (Tyr416), anti-pLyn (Tyr396) antibodies with anti-Src and anti-Lyn as their respective loading controls and anti-PSD95 and anti-synaptophysin with βIII-tubulin as the loading control. Antibody binding was detected using enhanced chemiluminescence for detection. Western blots were quantified using Adobe Photoshop software. Optical density (O.D.) of bands were normalized against their respective loading controls and averaged (+/−SEM).

### T-maze

T-maze analysis was performed as described by Wenk, 1998 [56]. Briefly, upon completion of the *in vivo* infusion period, control and treated mice were placed into the starting arm, and the door was raised to allow animals to walk down the stem and choose an arm. Once the mice entered an arm with all four feet, they were returned to the starting arm and the door was closed. After 30 sec, the door was opened and the mice were allowed to choose an arm again. The process was repeated for nine trials with a 30 sec interval between each trial. The choice of arms was noted each time and the number of alternations between trials for each mouse was averaged and plotted.

### Statistical analysis

Data are presented as mean +/− standard deviation. Values statistically different from controls were determined using one-way ANOVA or Student *t*-test when appropriate. The Tukey-Kramer multiple comparisons *post-hoc* test was used to determine *P*-values.

## Results

### The non-receptor tyrosine kinase inhibitor, dasatinib, decreased active-phospho Src levels in microglia BV2 cells

**Figure 1:**
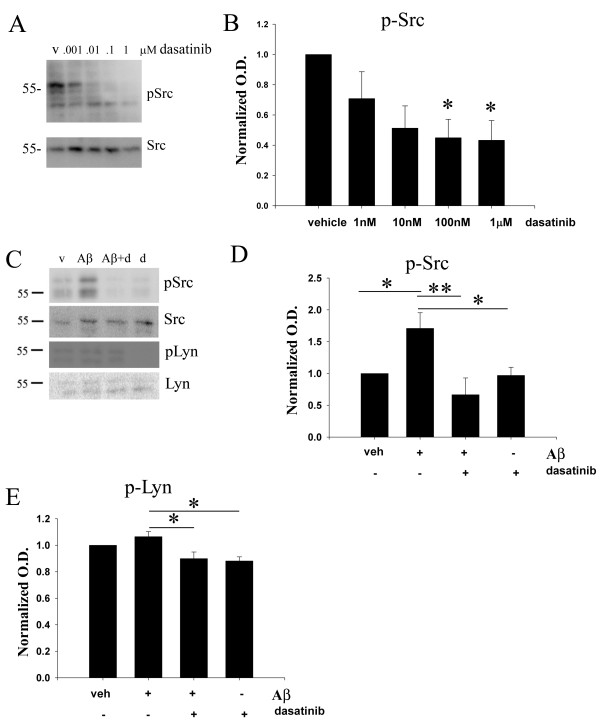
**Dasatinib dose-dependently attenuated active, phospho-Src kinase levels in the BV2 cell line and primary microglia.** (**A**) Microglial BV2 cells were vehicle treated (v), or treated with 1 nM, 10 nM, 100 nM and 1 μM dasatinib for 30 minutes. Cells lysates were resolved by SDS-PAGE and Western blotted using anti-phospho-Src, and Src antibodies. (**B**) Densitometric analyses of the Western blots were performed normalizing active-Src levels against their respective Src controls and averaging +/−SEM. Blots are representative of six independent experiments**.** Primary microglia were DMSO vehicle treated (veh), or stimulated for 5 minutes with 10 μM Aβ_f_ in the presence or absence of 30-minute pretreatment of 100 nM dasatinib. (**C**) Cells lysates were Western blotted using anti-pSrc or Src (loading control) antibodies and anti-pLyn or Lyn (loading control) antibodies. A representative blot from six independent experiments is shown. Densitometric analyses of the Western blots was performed normalizing (**D**) active, phospho-Src levels against Src controls and (**E**) active, phospho-Lyn levels against Lyn controls and averaging +/−SEM. Percent fold changes in phospho-Src and phospho-Lyn levels were plotted. (*P <0.05 vs. vehicle, dasatinib, **P <0.01 vs. Aβ + dasatinib for pSrc and *P <0.01 vs. Aβ for pLyn).

Based upon our prior work demonstrating that both fibrillar and oligomeric Aβ stimulate increased non-receptor tyrosine kinase activity in microglia [38], we determined whether a brain penetrant tyrosine kinase inhibitor, dasatinib, could attenuate activity of Src kinase *in vitro*. In order to validate the Src-kinase inhibitory ability of dasatinib, the mouse microglia cell line, BV2, was treated with varying concentrations of the drug. Dasatinib is a small molecule ATP-competitive inhibitor of Bcr-Abl and Src kinase with IC50s for the isolated kinases of 0.55 and 3.0 nM, respectively [57,58]. FDA-approved for use in cases of chronic phase Philadelphia chromosome-positive chronic myelogenous leukemia (CP-CML), dasatinib (Sprycel, Bristol-Myers Squibb, Princeton, NJ, USA) has been shown to cross the blood-brain barrier [59]. Although dasatinib will certainly attenuate c-Abl activity, based upon limited demonstration of c-Abl in microglia [60] and our prior work demonstrating robust Aβ-stimulated increase in Src family kinase activity [38,41], we expected a major target of dasatinib inhibition to be the Src family of non-receptor tyrosine kinases. As expected, active, phosphorylated Src levels significantly decreased with increasing dasatinib concentrations from 1nM to up 1 μM compared to vehicle treated BV2 cells (Figure 1). For subsequent treatments of microglial cells *in vitro*, a dose of 100 nM dasatinib was chosen to obtain an optimum effect of Aβ-dependent tyrosine kinase inhibition in primary cultures.

### Dasatinib attenuated the Aβ-stimulated increase in active-phospho Src levels in primary murine microglia cultures

In order to determine whether dasatinib was able to reduce an Aβ stimulated increase in active tyrosine kinase levels, primary microglia cultures were treated with Aβ fibril with or without dasatinib. Microglial cells were pretreated with 100 nM dasatinib followed by a five-minute stimulation by 10 μM Aβ_f_ to detect changes in the activated, phosphorylated forms of the specific non-receptor tyrosine kinases, Src and Lyn. As expected, Aβ fibrils stimulated an increase in active phospho-Src levels compared to vehicle treated cells (Figure 1). The Aβ-dependent increase in phospho-Src levels was significantly attenuated when cells where stimulated in the presence of dasatinib (Figure 1). Although Aβ fibrils did not significantly increase the levels of active, phosphorylated Lyn kinase at the concentrations used, dasatinib was still able to significantly reduce levels of active phospho-Lyn indicating that the drug was not specific to any particular Src family member (Figure 1). In addition, the data demonstrated that the Aβ stimulated increase in microglial active Src kinase levels could be inhibited using a clinically relevant non-receptor tyrosine kinase Src/Abl inhibitor, dasatinib.

### Dasatinib attenuated Aβ-stimulated TNF-α secretion in primary microglia cultures

**Figure 2:**
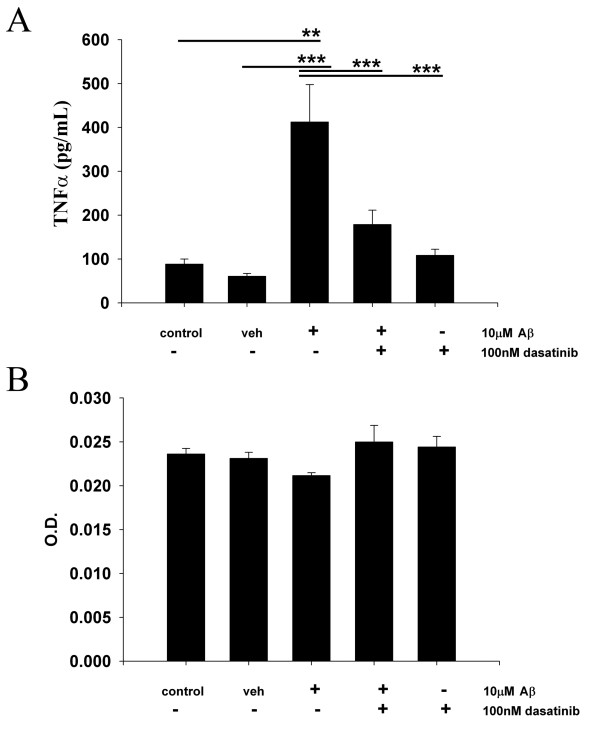
**Dasatinib attenuated Aβ1-42 stimulated-microglial secretion of the proinflammatory cytokine, TNF-α.** Primary microglia were unstimulated (control), DMSO vehicle treated (veh), or stimulated for 24 hours with 10 μM Aβ_f_ in the presence/absence of 100 nM dasatinib. (**A**) Media was collected from treated cells and used to quantify changes in TNF-α secretion via ELISA. Secreted values were averaged +/−SEM. (**B**) After media removal, the treated cells were used to assess cell viability via the MTT reduction assay. Absorbance values (560/650 nm) were averaged+/−SEM. (**P <0.01 from control, ***P <0.001 vs. vehicle, dasatinib and Aβ + dasatinib).

In order to determine whether the Aβ-stimulated change in active Src levels was required for changes in phenotype, changes in secretion of the pro-inflammatory cytokine, TNF-α, were quantified from the primary microglia cultures in the presence or absence of dasatinib. Fibrillar Aβ stimulated a significant increase in TNF-α secretion compared to control or vehicle treated microglial cells (Figure 2). This increase in TNF-α secretion was attenuated by 100nM pretreatment of dasatinib with no effect of cellular viability (Figure 2). The data demonstrated that inhibition of Src family kinase activity was sufficient to prevent microglia from acquiring a reactive secretory phenotype upon Aβ fibril stimulation in vitro.

### An age-dependent increase in Aβ plaque density correlated with microgliosis in a transgenic mouse model of AD

**Figure 3:**
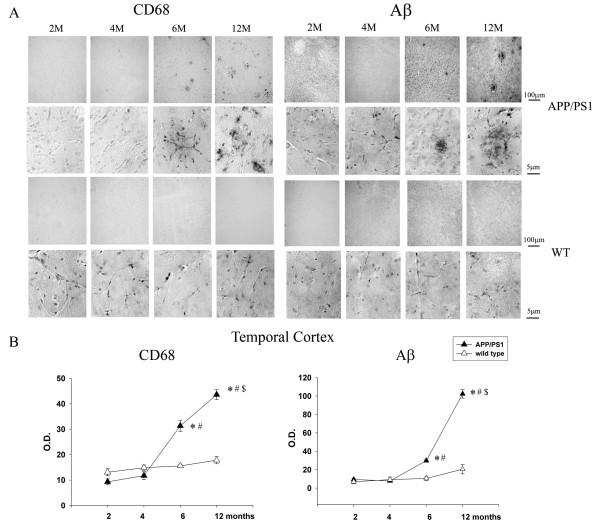
**Microgliosis in the APP/PS1 model of AD positively correlated with increased Aβ plaque density with age.** Serial coronal sections from 2- , 4- , 6- and 12-month old controls (C57BL/6) and age-matched transgenic animals (APP/PS1) (n = 5 to 6) were immunostained for (**A**) activated microglia (anti-CD68) and Aβ (anti-4G8 antibody). Representative hemi-sections are shown with select high magnification fields. (**B**) Immunoreactivity densities from the temporal cortex region for CD68 and 4G8 were quantified, averaged and graphed (+/−SEM) for *P <0.001 from 2- and 4-month APP/PS1 animals, ^#^*P* <0.001 from 2-, 4-, 6- and12-month wild type animals for CD68, ^#^*P* <0.001 from 2-, 4- and 6-month wild type mice for 4 G8, and ^$^*P* <0.001 from 6-month APP/PS1 animals.

**Figure 4:**
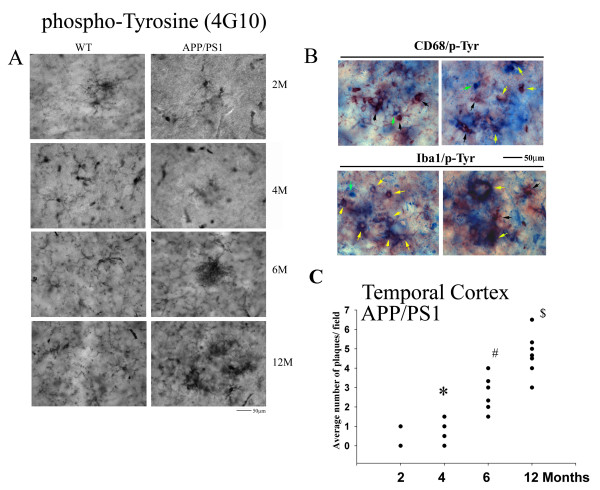
**Plaque-associated microglial phosphotyrosine immunoreactivity increased with age in APP/PS1 mice.** (**A**) Serial coronal sections from 2- , 4- , 6- and 12-month-old controls (C57BL/6) and age-matched transgenic animals (APP/PS1) (n = 5 to 6) were immunostained with anti-phosphotyrosine antibody (4G10). Representative select high magnification fields from the temporal cortex of hemisections are presented. (**B**) To validate microglial co-localization of phospho-tyrosine immunoreactivity, 12-month APP/PS1 sections were double-labeled using anti-phosphotyrosine antibodies and VIP as the chromogen (black arrows) and anti-CD68 or Iba1 and Vector Blue as the chromogen (green arrows). Double-labeled cells are indicated with yellow arrows. (**C**) To quantify immunoreactivity and omit the inclusion of non-microglial phosphotyrosine immunoreactivity, 4G10 positive plaques from APP/PS1 mice were counted from the temporal cortex and averaged and graphed (+/−SEM) for *P <0.001 from two-month APP/PS1 mice, ^#^*P* <0.001 from two-month and four-month APP/PS1 animals, ^$^*P* <0.001 from four- and six-month APP/PS1 animals.

To determine an appropriate age *in vivo* for examining increased tyrosine kinase activity and Aβ-associated microgliosis, a transgenic APP/PS1 mouse model of AD was used. Varying age brains of APP/PS1 mice were compared to age-matched controls to validate the use of this transgenic mouse model for our study. The APP/PS1 mice were collected at 2, 4, 6 and 12 months of age along with their age-matched wild type mice (C57BL/6) and brains were sectioned for histology. Immunostaining demonstrated a significant increase in APP/PS1 temporal cortex CD68 immunoreactivity in 6- and 12-month old mice compared to earlier 2- and 4-month APP/PS1 age groups and their age-matched C57BL/6 controls (Figure 3). This increase correlated precisely with increased plaque-associated Aβ immunoreactivity in the 6- and 12-month old APP/PS1 mice (Figure 3). Analyses of hippocampi from all groups showed similar results to the temporal cortex (data not shown). These data supported the notion that microglial activation in AD and its mouse models may be a consequence of Aβ fibril interaction. However, to validate an involvement of tyrosine kinase activity in microglial phenotype changes during disease, APP/PS1 brains were next immunostained with anti-phosphotyrosine antibody as an indirect method to assess overall tyrosine kinase activity changes. Immunostaining mouse brains with anti-phosphotyrosine antibody, 4G10, demonstrated positive microglial-like as well as vascular immunoreactivity (Figure 4). Microglial-like immunoreactivity increased in intensity demonstrating transition from ramified to a more compact, plaque-clustered morphology with increasing age in APP/PS1 mice compared to controls (Figure 4). Counting plaque-associated, 4G10 positive staining as putative microglia demonstrated a trend of age-associated increase in APP/PS1 temporal cortex staining with a significant increase by 4 months of age with significantly higher immunoreactivity at 6 and 12 months (Figure 4). Similar trends were observed in the hippocampus (data not shown). To validate that phosphotyrosine immunoreactivity partially co-localized to microglia, sections were double-labeled with both antiphosphotyrosine antibody and anti-CD68 or anti-Iba1 antibodies. Double-labeling demonstrated that a portion of the phosphotyrosine immunoreactivity demonstrated clear co-localization with either microglial marker, CD68 or Iba1 (Figure 4). These data confirmed that by 12 months of age, this particular transgenic line had robust phosphotyrosine microglial reactivity in association with Aβ plaques providing us with the appropriate time point for use.

### Dasatinib infusion decreased phospho-Src but not phospho-Lyn levels in APP/PS1 mice

**Figure 5:**
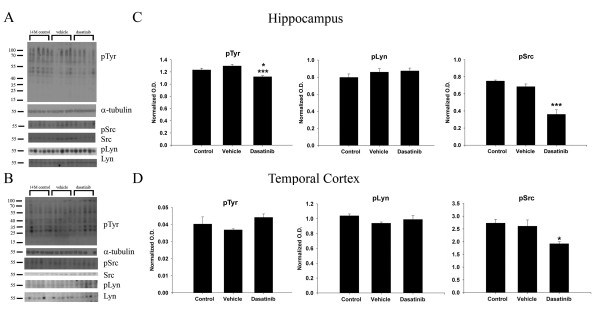
**Dasatinib infusion reduced protein phospho-tyrosine and active phospho-Src kinase levels in APP/PS1 brains.** Vehicle or dasatinib (500 ng/kg/day) was infused subcutaneously into 13-month-old APP/PS1 for 28 days (n = 7). Hippocampus and temporal cortex regions were dissected from the left hemispheres and resolved by 10% SDS-PAGE and Western blotted. (**A**) Hippocampal and (**B**) temporal cortex brain lysates from control (14-month APP/PS1), vehicle infused, and dasatinib infused animals were blotted using anti-phosphotyrosine, pTyr (4G10), pSrc, pLyn, α-tubulin, Src, and Lyn antibodies. Optical densities were averaged and graphed (+/−SEM) from blots of (**C**) hippocampus (*P <0.05 from controls, ***P <0.001 from vehicle) and (**D**) temporal cortex. (*P <0.05 from controls).

Based upon the temporal profiling of microgliosis we next determined whether inhibition of tyrosine kinase activity could attenuate the microglial reactivity in this line. Based upon our in vitro data we hypothesized that dasatinib treatment would attenuate the Aβ-associated increase in tyrosine kinase activity, particularly Src, and subsequent inflammatory changes in the mice. In order to answer this question, 13 month old APP/PS1 female mice were subcutaneously infused with dasatinib or vehicle for 28 days. Dasatinib infusion significantly decreased protein phosphotyrosine levels in the hippocampus but not the temporal cortex compared to vehicle infused control animals (Figure 5). More importantly, dasatinib infusion significantly decreased levels of active, phospho-Src in both temporal cortex and hippocampus compared to control mice (Figure 5). However, dasatinib infusion had no effect on the active, phosphorylated levels of the related Src family member, Lyn kinase, demonstrating some specificity of the drug (Figure 5). These data demonstrated that a subcutaneous route of dasatinib delivery was able to inhibit levels of active Src in the brains of the APP/PS1 mice.

### Dasatinib infusion decreased TNF-α levels and microgliosis in APP/PS1 mice

**Figure 6:**
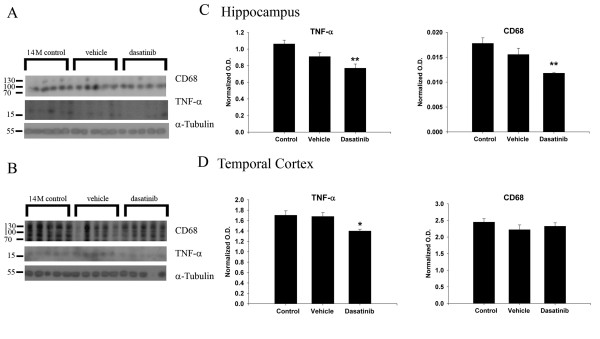
**Dasatinib infusion reduced CD68 and TNF-α protein levels in APP/PS1 brains.** (**A**) Hippocampal and (**B**) temporal cortex brain lysates from control, vehicle, and dasatinib infused animals were Western blotted for activated microglia using anti-CD68 antibody and anti-pro-inflammatory cytokine TNF-α antibody along with anti-α-tubulin as a loading control. Optical densities were averaged and graphed (+/−SEM) from blots of (**C**) hippocampus (**P <0.01 from controls) and (**D**) temporal cortex (*P <0.05 from controls).

Based upon the encouraging findings that dasatinib was able to exert brain effects and decrease active Src levels, we expected that dasatinib infusion should also attenuate microgliosis and TNFα secretion as observed in our *in vitro* findings. Dasatinib infusion significantly decreased TNFα levels in both the hippocampus and temporal cortex correlating with the decrease in active Src levels and demonstrating a clear brain anti-inflammatory effect of the drug (Figure 6). Moreover, levels of CD68, the reactive microglial marker protein, were significantly decreased in the hippocampus of dasatinib infused brains but not the temporal cortex. These data demonstrated that dasatinib inhibition of brain active Src levels correlates with a significant anti-inflammatory, microglial-inhibitory effect in APP/PS1 mice. Interestingly, these effects appeared most robust in the hippocampus compared to the temporal cortex (Figure 6).

### Dasatinib infusion did not alter APP, Aβ, or synaptic protein levels in APP/PS1 mice

**Figure 7:**
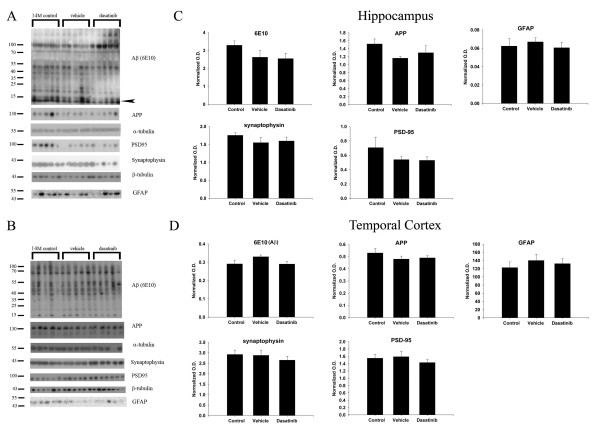
**Dasatinib infusion did not alter levels of APP, Aβ, synaptophysin, PSD95, or GFAP levels in APP/PS1 brains.** (**A**) Hippocampal and (**B**) temporal cortex brain lysates from control, vehicle, and dasatinib infused animals were used for Western blot analyses using anti-Aβ clone 6E10 antibody, anti-APP, anti-synaptophysin, anti-PSD95, anti-GFAP, anti-α-tubulin and βIII tubulin (loading control) antibodies. Optical densities for each antibody were normalized to tubulin loading controls, averaged, and graphed (+/−SEM) for (**C**) hippocampus and (**D**) temporal cortex.

To examine the breadth of changes that might result from dasatinib infusion we also determined whether adverse effects on neurons, astrocytes, or Aβ deposition resulted from drug treatment in addition to the anti-inflammatory, microglial-inhibitory changes. However, dasatinib administration had no effect on either total APP levels or Aβ levels in the hippocampus or temporal cortex of treated mice compared to vehicle controls (Figure 7). In addition, dasatinib treatments did not increase levels of the reactive astrocyte marker protein, GFAP, in the infused animals compared to vehicle control mice (Figure 7). Finally, dasatinib treatment did not have any significant effects on levels of either presynaptic, synaptophysin, or post-synaptic, PSD 95, proteins in either the temporal cortex or hippocampus compared to vehicle treated mice (Figure 7). These data demonstrated that the Src inhibitory, anti-inflammatory, microglia-inhibitory effects of dasatinib treatment did not correlate with any robust adverse effects such as increased astrogliosis, neuron or synaptic loss, and increased Aβ deposition.

### Dasatinib infusion decreased immunoreactivity of protein phosphotyrosine, phospho-Src and CD68 in APP/PS1 mice

**Figure 8:**
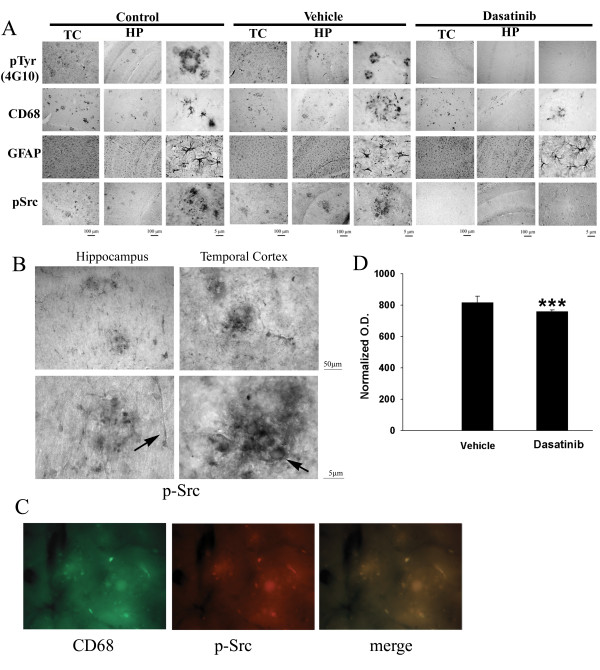
**Dasatinib infusion attenuated microgliosis, protein phosphotyrosine and active phospho-Src immunoreactivity with no effect on GFAP immunoreactivity in APP/PS1 brains.** Right hemispheres from brains of dasatinib infused, vehicle infused and APP/PS1 controls animals were fixed, serially sectioned and immunostained using (**A**) anti-phosphotyrosine (4 G10), anti-CD68, anti-GFAP, and anti-phospho-Src (pSrc) antibodies. Representative images from the CA1 region of the hippocampus (HP) as well as the temporal cortex (TC) are shown with select high magnification TC fields. (**B**) High magnification images of pSrc immunoreactivities from control APP/PS1 temporal cortex and hippocampus demonstrate robust plaque-associated immunoreactivity (arrow) along with vascular immunoreactivity (arrow). (**C**) Fluorescent double-labeling of control APP/PS1 brains demonstrated clear pSrc (red) immunoreactivity co-localizing with CD68 (green) staining. (**D**). Relative levels of pSrc immunoreactivity in the temporal cortex were quantified from serial stained sections from vehicle and dasatinib infused brains, averaged (+/−SEM), and graphed (***P <0.001 via student’s *t*-test).

To provide qualitative assessments along with the quantitative Western blot analyses, select immunohistochemical studies were also performed from the treated animals. As expected, protein phosphotyrosine immunoreactivity in dasatinib infused mouse brains appeared dramatically less than vehicle treated mice in both the hippocampus and temporal cortex (Figure 8). A similar trend of dasatinib decreased immunoreactivity was observed for active phospho-Src versus vehicle controls (Figure 8). Similar to phosphotyrosine, pSrc immunoreactivity also localized to vasculature as well as microglial-like immunoreactivity (Figure 8). Double-labelling verified that a portion of the pSrc immunoreactivity co-localized to microglia (Figure 8). Because the majority of pSrc immunoreactivity was observed in plaque-associated microglial-like cells we suggest that the significant decreases in active Src and phosphotyrosine levels observed by Western blot analyses were a reflection of microglial changes due to the drug. However, certainly a portion of both phosphotyrosine and pSrc level changes detected by the Western blot analyses is due to contributions from cells other than microglia. Also as predicted, levels of CD68 reactive microglial immunoreactivity in dasatinib infused mice was noticeably decreased compared to vehicle treated mice in both the hippocampus and temporal cortex (Figure 8). Consistent with the Western blot analysis, GFAP immunoreactivity for reactive astrocytes was not visually different between dasatinib and vehicle infused mice in either temporal cortex or hippocampus (Figure 8). The qualitative immunostaining findings corroborated the quantitative Western blot analyses and suggest that the significant decreases in pSrc levels were largely due to decreased microglial immunoreactivity, our intended target for the drug.

### Aβ plaque load was not affected by dasatinib infusions into the APP/PS1 mice

**Figure 9:**
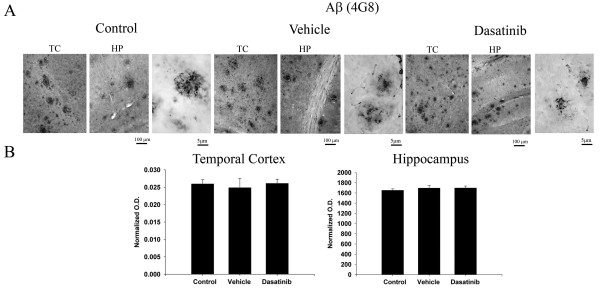
**Dasatinib infusion did not affect Aβ plaque load in the brains of APP/PS1 mice.** Serial sections of right hemispheres from brains of dasatinib infused, vehicle infused, and control APP/PS1 mice were immunostained using anti-Aβ antibody (clone 4 G8) and plaque load was quantified. (**A**) Representative images of the temporal cortex (TC) and hippocampus (HP) from brain hemispheres are shown with select high magnification TC fields. (**B**) Mean optical density measurements from the CA1 region of the hippocampus as well as the temporal cortex are shown +/− SEM.

One possible consequence of attenuated tyrosine kinase activity in microglia could be inhibition of phagocytic ability. It is well known, for instance, that tyrosine kinase activity can affect macrophage phagocytic ability [61]‐[63]. Therefore, inhibition of Src or related kinase activity might attenuate any ability microglia have to clear Aβ deposits in the brain producing an unwanted side-effect of increased plaque load. Based upon the Western blot analysis, dasatinib treatment had no effect on altering APP or Aβ levels in the brains of mice (Figure 8). However, to better assess whether the drug had any effects on insoluble fibrillar deposits in the brain, quantitative immunostaining of Aβ plaques was performed. Dasatinib treatment had no effect on plaque load in treated mice compared to vehicle controls in either the hippocampus or temporal cortex (Figure 9). These data demonstrate that the microglial-inhibitory effects of dasatinib do not result in unwanted effects of increasing Aβ deposition or accumulation.

### Dasatinib infusion improved T maze performance in APP/PS1 mice

**Figure 10:**
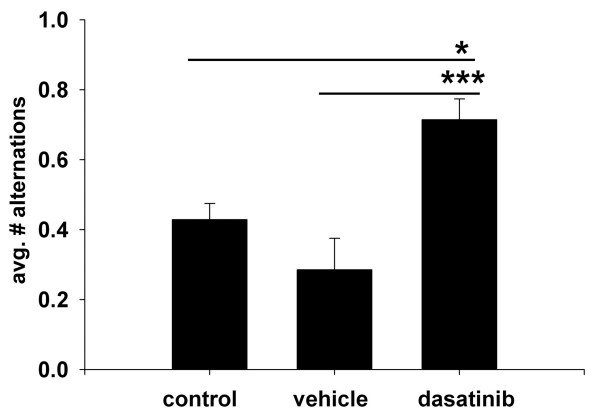
**Dasatinib infused APP/PS1 mice demonstrated increased spontaneous alternations during T maze testing.** Control untreated 14-month APP/PS1 mice, dasatinib infused mice or vehicle infused controls (n = 7) were used for T maze testing on Day 29 following 28 days of drug or vehicle infusion. The mean number of spontaneous alternations +/− SEM per treatment group were graphed. (*P <0.05 vs. control, ***P <0.001 vs. vehicle).

Although there was no effect of dasatinib treatment on Aβ deposition, the clear and somewhat specific effect of Src inhibition correlating with anti-inflammatory, microglial-inhibitory effects provided an opportunity to assess whether dasatinib could also provide cognitive improvement to the mice. After treatments, spontaneous alternations from dasatinib treated, vehicle treated and control animals were quantified during T maze analyses. This particular model of spatial memory was selected based upon its sensitivity of detection of cognitive deficit in AD mouse models [64] and its preference for detecting changes in hippocampal-based performance differences [65,66] since our most significant effects of drug were observed in the hippocampus. Dasatinib infused animals demonstrated improved cognitive performance represented by a significant increase in spontaneous alternations compared to vehicle treated mice (Figure 10). These data validated that subcutaneous administration of the Src/Abl tyrosine kinase inhibitor, dasatinib, is able to provide cognitive enhancing effects to APP/PS1 mice while attenuating microglial activation and proinflammatory cytokine, TNFα, levels in the brain.

## Discussion

Our findings demonstrated that Aβ fibrils stimulate microglia activation in vitro via a non-receptor tyrosine kinase, Src, associated pathway that results in increased secretion of the proinflammatory cytokine, TNF-α. It was possible to attenuate the Aβ-stimulated microglial phenotype change using a dual Src/Abl inhibitor, dasatinib. The in vitro observations were validated in vivo demonstrating that subcutaneous infusion of dasatinib into 13 month old APP/PS1 transgenic mice attenuated overall tyrosine phosphorylation and active Src levels, in particular, in the hippocampus. The drug did not affect Aβ plaque load but reduced microgliosis and TNF-α levels in these animals without altering synaptic markers in neurons. Moreover, dasatinib provided a significant increase in cognitive performance in correlation with this anti-inflammatory action. Collectively, these findings support the idea that Aβ fibrils can serve as a microglial activating ligand in disease contributing to their proinflammatory phenotype and use of selective non-receptor tyrosine kinase inhibitors is an effective strategy to limit microglial-mediated changes during disease.

Although it has been suggested that Aβ-stimulated microglial activation contributes to the pathophysiology of AD [24]‐[26,28]‐[30,33,67] and a broad range of microglial secreted inflammatory markers are elevated in AD brains, including IL-1α, IL-1β, TGF-β and TNF-α [68,69], enthusiasm for an anti-inflammatory approach to treating AD has decreased, in part, due to lack of drug efficacy of a number of human trials that targeted cyclooxygenase (Cox) activity in AD patients [70]‐[74]. Indeed, Cox inhibition during later stages of disease had adverse effects and only demonstrated protection when administered long-term to asymptomatic individuals [37]. One possibility for the lack of efficacy of Cox inhibitors is the fact that Cox enzymes are expressed by multiple cell types in the brain and general drug inhibition has no cellular selectivity. Another possibility for the failed efficacy is that Cox 1 or 2 enzyme activities are simply not relevant targets for attenuating microglia-dependent changes. For this reason we focused on the direct signaling response initiated in microglia upon Aβ fibril stimulation. It has been reported from both AD brains [75]‐[77] and mouse models [13,78] that elevated protein phosphotyrosine levels are reliable markers of reactive microglia associated with plaques. *In vitro* studies using monocytic lineage cells [24,44,47] and microglia [38,41] have supported these data by demonstrating that fibrillar Aβ stimulates a specific increase in overall protein tyrosine phosphorylation. Based upon our *in vitro* data, we targeted Src as a key enzyme activated downstream of Aβ fibril stimulation and demonstrated that a clinically available drug, dasatinib, was able to improve cognitive function while attenuating microglial activation and active Src levels in these cells. Importantly, this anti-inflammatory effect did not adversely affect Aβ plaque load in the mice. Therefore, a directed anti-inflammatory strategy targeting the particular enzymes involved in Aβ-stimulated microgliosis may be more relevant than broad-based Cox inhibition for testing during disease. Moreover, the fact that this tyrosine kinase inhibition strategy was effective even during advanced stages of disease in the mice suggests that this particular form of anti-inflammatory therapy is viable during advanced disease in contrast to Cox inhibition.

We are aware that the effect of decreasing microglial active Src and brain TNFα levels does not necessarily prove that these changes were responsible for the improved cognitive performance. However, our in vitro data clearly demonstrated that dasatinib treatment and Src inhibition led to attenuated TNFα secretion providing correlative evidence that Src inhibition in microglia in vivo contributed to the decrease in TNFα observed. Moreover, recent human data using TNFα neutralizing drugs demonstrated cognitive improvement in AD patients [79] suggesting that diminished TNFα levels in the mice could have contributed to the cognitive improvements observed.

We also appreciate that dasatinib treatment may affect a number of other kinases *in vitro* and *in vivo,* and numerous cells express Src family kinases and Abl. Non-receptor Src family tyrosine kinases are expressed widely in the mammalian CNS and are known to play a role in proliferation and differentiation of the CNS [80]‐[89]. Indeed, Src family kinase activities are crucial for synaptic plasticity, including learning and memory [90]‐[94]. Additionally, there is compelling evidence that neuronal Abl activity can also mediate microgliosis *in vivo,* suggesting that dasatinib may also work through this mechanism to exert its anti-inflammatory effects [60]. It is also intriguing that Abl is able to phosphorylate tau protein on Tyr 394 identified from AD brains as well as the intracellular domain of APP to modulate signaling responses [95]‐[97]. Although we have not focused on Abl expression or activity in this study we appreciate that activity of this kinase is of great interest to not only the field of AD but myriad conditions in which oxidative stress-associated neuron death is involved, including Parkinson’s disease [60,96,98]‐[102]. However, this interest in Abl activity to regulate cell death [60], parkin phosphorylation [99,100] and tau phosphorylation [98,101,102] is all based upon neuronal expression of the kinase. Indeed, there is no reported expression of Abl in microglia, to the best of our knowledge. We expect that dasatinib actions in the brain will certainly include inhibition of not only microglial Src activity but additional Src family members expressed in microglia and other cells as well as Abl, which will have a broader target base than only a single kinase activity in microglia. This may have additional therapeutic benefits of not only anti-inflammatory actions on microglia but also direct neuroprotection. We do not rule out that some of the changes we observed are not also due to inhibition of Src family kinases or other non-receptor kinases, including Abl in our experiments. Certainly, our staining demonstrated additional vasculature phospho-tyrosine and pSrc immunoreactivity outside of the microglial immunoreactivity. Therefore, any strategy to manipulate activity of these tyrosine kinases in the brain should be carefully considered with regard to particular cellular targets. By focusing on microglia-Aβ interaction and demonstrating specificity of dasatinib for Src versus the related family member Lyn *in vivo* as well as a clear improvement in cognitive performance, we suggest that reagents, such as dasatinib, at least be considered for anti-inflammatory human testing but, more importantly, for further drug development.

Our study intentionally focused on animals at 13 months of age with established plaques and reactive microglia to test the efficacy of our anti-inflammatory strategy in late-stage disease. However, it will be important in future work to determine if a strategy of kinase inhibition can attenuate or delay behavioral decline or microgliosis in earlier stage disease. Although our longitudinal assessment in these mice suggested that phosphotyrosine immunoreactive microglia correlated with increased plaque deposition, we have observed in earlier work that soluble oligomeric forms of Aβ are also potent stimuli of microglia responsible for initiating a unique type of tyrosine kinase-based signaling response [38]. Therefore, fully determining the specific signaling pathways involved in different forms of Aβ stimulation of microglia may offer a strategy for inhibiting specific tyrosine kinase activities at different disease time points to maximally produce anti-inflammatory effects.

## Conclusions

These data demonstrate that the mechanism underlying amyloid-dependent microgliosis in AD may involve increased Src family kinase activity. We targeted this specific signaling response with dasatinib, a dual Src/Abl inhibitor used for treatment of chronic myeloid leukemia [103,104] For the first time, we have found that dasatinib treatment not only attenuated microgliosis, TNFα levels, and active Src levels in the brains of APP/PS1 mice but also improved cognitive performance. This suggests that targeting the specific enzymes involved in Aβ-stimulated microglial activation may be efficacious therapeutically even during late stages of disease.

AD, Alzheimer’s Disease; Aβ, amyloid beta; APP, amyloid precursor protein; GFAP, glial fibrillary acidic protein; Iba1, ionized calcium binding adaptor molecule 1; PSD95, postsynaptic density protein 95; TNFα, tumor necrosis factor α.

## Competing interests

The authors declare that they have no competing interests.

## Authors’ contributions

GD performed the majority of experiments and data analysis, and wrote the initial version of the manuscript. CC was involved in conceiving the study, performing a portion of the experiments and analysis, and coordinating the experiments. He was responsible for editing and revising the final version of the manuscript. All authors read and approved the final manuscript.
